# Risk factors of early pulmonary hypertension and its clinical outcomes in preterm infants: a systematic review and meta-analysis

**DOI:** 10.1038/s41598-022-18345-y

**Published:** 2022-08-19

**Authors:** Yoo Jinie Kim, Seung Han Shin, Hye Won Park, Ee-Kyung Kim, Han-Suk Kim

**Affiliations:** 1 Division of Neonatology, Department of Pediatrics, Konkuk University Medical Center, Seoul, Korea; 2 Department of Pediatrics, Seoul National University College of Medicine, 101, Daehak-ro, Jongno-gu, Seoul, 03080 Korea; 3 Division of Neonatology, Department of Pediatrics, Seoul National University Children’s Hospital, Seoul, Korea; 4 Department of Pediatrics, Konkuk University School of Medicine, Seoul, Korea

**Keywords:** Diseases, Health care, Medical research, Risk factors, Signs and symptoms

## Abstract

The aim of this meta-analysis was to determine the incidence and risk factors of early pulmonary hypertension (PHT) in preterm infants and evaluate the association of early PHT with morbidities such as bronchopulmonary dysplasia (BPD), late PHT, and in-hospital mortality. We searched the PubMed (1980–2021), Embase (1968–2021), CINAHL (2002–2021), Cochrane library (1989–2021), and KoreaMed (1993–2021). Observational studies on the association between early PHT diagnosed within the first 2 weeks after birth and its clinical outcomes in preterm infants born before 37 weeks of gestation or with very low birth weight (< 1500 g) were included. Two authors independently extracted the data and assessed the quality of each study using a modified Newcastle–Ottawa Scale. We performed meta-analysis using Comprehensive Meta-Analysis version 3.3. A total of 1496 potentially relevant studies were found, of which 8 studies (7 cohort studies and 1 case–control study) met the inclusion criteria comprising 1435 preterm infants. The event rate of early PHT was 24% (95% confidence interval [CI] 0.174–0.310). The primary outcome of our study was moderate to severe BPD at 36 weeks postmenstrual age, and it was associated with early PHT (6 studies; odds ratio [OR] 1.682; 95% CI 1.262–2.241; *P* < 0.001; heterogeneity: *I*^2^ = 0%; *P* = 0.492). Preterm infants with early PHT had higher OR of in-hospital mortality (6 studies; OR 2.372; 95% CI 1.595–3.528; *P* < 0.001; heterogeneity: *I*^2^ = 0%; *P* = 0.811) and developing late PHT diagnosed after 4 weeks of life (4 studies; OR 2.877; 95% CI 1.732–4.777; *P* < 0.001; heterogeneity: *I*^2^ = 0%; *P* = 0.648). Infants with oligohydramnios (4 studies; OR 2.134; 95% CI 1.379–3.303; *P* = 0.001) and those who were small-for-gestational-age (5 studies; OR 1.831; 95% CI 1.160–2.890; *P* = 0.009) had an elevated risk of developing early PHT. This study showed that early PHT is significantly associated with mortality and morbidities, such as BPD and late PHT. Preterm infants with a history of oligohydramnios and born small-for-gestational-age are at higher risk for developing early PHT; however, high-quality studies that control for confounders are necessary.

## Introduction

Advances in perinatal and neonatal medicine have led to a steady increase in the survival of preterm infants. However, the incidence of chronic lung disease and its associated complications in this population have not decreased over past few decades^1^. This might be attributed to the increased rate of survival of immature and extremely preterm infants. The survival of extremely preterm infants could interrupt the developmental process of the premature lung, represented by alveolar simplification and dysmorphic vascular growth^2^. Early disruption of angiogenesis in the developing lung can impair alveolarization and cause structural and functional abnormalities^3–5^. Recently, the concept of pulmonary vascular disease in preterm infants has been utilized for understanding the association between impaired development of immature lungs and comorbid conditions, such as bronchopulmonary dysplasia (BPD) and pulmonary hypertension (PHT), in preterm infants. The incidence of PHT in patients with BPD, which is evaluated at discharge or 36 weeks postmenstrual age, ranges from 4 to 33% depending on the severity of BPD^6^. Recent studies have demonstrated that preterm infants with BPD-associated PHT have a high mortality rate of up to 38%^7,8^. In addition, several morbidities, such as long duration of respiratory support and poorer cognitive functions, are correlated with late PHT^9–11^.

Given that both BPD and PHT originate from disruption of the development of the lung, pulmonary vascular disease could also manifest earlier in life as PHT. PHT in the early postnatal period occurs in 3%–42% of extremely preterm infants. Mourani et al. reported that early PHT was identified in 42% of extremely preterm infants and was associated with increased severity of BPD and late PHT^12^. In a recent retrospective cohort study, 30% of extremely preterm infants were diagnosed with early PHT^13^. In addition, the population showed an increased rate of death and BPD-associated PHT. The results of several single-center studies have suggested that the risk factors of early PHT include preterm birth, being small-for-gestational-age, and oligohydramnios^13–15^. However, the etiology and clinical burden of pulmonary vascular disease in early life are still unclear.

This study aimed to investigate the incidence and association of early PHT with mortality, morbidities such as BPD, and late PHT. Furthermore, we analyzed risk factors of early PHT in preterm infants through the systematic review and meta-analysis of previous studies on early PHT.

## Methods

### Study design

This systematic review of the literature and meta-analysis was conducted according to the Meta-analysis of Observational Studies in Epidemiology (MOOSE) guidelines (Supplementary material)^16^. The protocol of this review is registered at PROSPERO (registration number: CRD42021269362 [http://www.crd.york.ac.uk/PROSPERO/]). Approval from an ethics board was not required since this was a systematic review of literature.

The PICO approach was used to formulate a search question as follows: P (population): study population composed of preterm or very low birth weight infants; I (intervention or exposure): participants experienced early PHT within 2 weeks of age; C (comparison): comparison of clinical outcomes and related risk factors in preterm infants who did not have early PHT; O (outcome): report one or more of the following outcomes: morbidity rate of BPD, PHT, and mortality.

### Data sources and search strategy

We searched the PubMed (1980–2021), Embase (1968–2021), CINAHL (2002–2021), Cochrane library (1989–2021), and KoreaMed databases (1993–2021) for relevant articles without placing any restrictions on language, population, or year of publication. The search terms were combinations of the following keywords: “preterm infant or premature infant or extremely low birth weight or very low birth weight,” “pulmonary hypertension or pulmonary arterial hypertension or pulmonary vascular disease,” “bronchopulmonary dysplasia or chronic lung disease,” and “mortality or death rate”. Review articles, case reports, editorials, and commentaries were excluded. Additional studies were manually searched from key articles that fulfilled our eligibility criteria by two authors (YJK and SHS). Endnote 20 (Clarivate Analytics, US) was used to merge citations of searched articles and for screening.

### Study selection

We included studies that satisfied the following criteria: (1) case–control study or prospective or retrospective cohort study; (2) study population included preterm (gestational age < 37 weeks) or very low birth weight (< 1500 g) infants with early PHT diagnosed within first 2 weeks of age who presented with clinical symptoms or showed relevant echocardiography results; and (3) study outcomes included BPD, late PHT, or mortality. Preterm infants with major congenital heart diseases (except patent ductus arteriosus, atrial septal defects, patent foramen ovale, or small ventricular septal defects) or other non-cardiac congenital anomalies were excluded in the study population. Studies that involved the evaluation of PHT among patients with BPD were eliminated. The titles and abstracts of the articles were first screened separately by two blinded reviewers (YJK and SHS). Thereafter, the full texts of the selected articles were also independently reviewed by two reviewers (YJK and SHS) to identify relevant studies that fulfilled our eligibility criteria. We used Cohen’s kappa coefficient (κ) to measure inter-reviewer reliability when selecting studies. The value of the coefficient (κ) less than 0.61 was considered as disagreement between two reviewers. Discrepancies were resolved through discussion and consultation with a third researcher (HSK).

### Exposures

The diagnostic criteria of early PHT were similarly documented and included at least one or more of the following echocardiographic findings: (1) estimated right ventricular systolic pressure (RVSP) > 40 mmHg, (2) RVSP/systemic systolic pressure > 0.5, (3) any cardiac shunt flow with bidirectional or right-to-left flow, or (4) ventricular septal wall flattening. Clinical diagnosis of early PHT included symptoms of hypoxemic respiratory failure and differential cyanosis. PHT assessed later than 2 weeks postnatal age was excluded.

### Outcomes

The primary outcome of the present study was moderate to severe BPD at 36 weeks postmenstrual age. BPD was defined as oxygen requirement for more than 28 days or need for oxygen supplementation at 36 weeks. The severity of BPD (mild, moderate, or severe) was determined at 36 weeks postmenstrual age using the criteria proposed by Jobe et al.^17^ with or without application of oxygen reduction test^18^. The secondary outcomes were clinical outcomes related to early PHT: in-hospital mortality and late PHT. Late PHT was defined as PHT diagnosed after more than 4 weeks of age or identified using echocardiography at or after 36 weeks postmenstrual age.

### Data extraction

Two researchers (YJK and SHS) independently extracted data from the selected articles. The data extracted from each article included first author, year of publication, study design, location where the study was performed, definitions of early PHT and BPD, characteristics of the study population, and results regarding each morbidity. In addition, information regarding in-hospital mortality were collected. We also extracted the number of subjects with prenatal history of oligohydramnios, preterm premature rupture of membrane (PPROM), chorioamnionitis, maternal history of preeclampsia, and who were born small-for-gestational age (SGA) to investigate related risk factors.

### Quality assessment

The methodological qualities of the studies were assessed by two researchers (YJK and SHS) using a modified Newcastle–Ottawa Scale for cohort or case–control studies^19^. This scale consists of three domains: selection (0–4 points), comparability (0–2 points), and exposure or outcome (0–3 points). Total scores of 0–3 were deemed to indicate a high risk of bias in a study, whereas scores of 4–6 and 7–9 indicate moderate quality and high quality, respectively. Disparities in the assessment were resolved through discussion with a third researcher (HSK).

### Data analysis

The meta-analysis was performed using Comprehensive Meta-Analysis version 3.3 (Biostat, Englewood, NJ, USA). Odds ratio (OR) with 95% confidence interval (CI) and *P*-value were calculated from the data provided in each study. A random-effects model was used to combine all study results. Statistical heterogeneity was assessed using the Cochrane Q statistic and Higgin’s *I*^2^ statistic. *I*^2^ statistic was derived from the Q statistic and described the percentage of the total variation across studies. *I*^2^ was calculated as (Q-df)/Q × 100%, where Q is the chi-squared statistic and df is its degree of freedom. *I*^2^ > 50% and a Q test of *P* < 0.1 was considered as having a significant heterogeneity. Sensitivity analysis was conducted to evaluate the robustness of the results by removing each study and repeatedly performing the meta-analysis on the pooled OR results. Although the meta-analysis included fewer than the recommended number of 10 studies, funnel plot was used to assess potential publication bias. We also performed the trim and fill method and contour-enhanced funnel plot to assess the possible effect of publication bias on asymmetry. The contour-enhanced funnel plot was performed with R software version 4.2.0.

## Results

### Literature search and study characteristics

**Figure 1:**
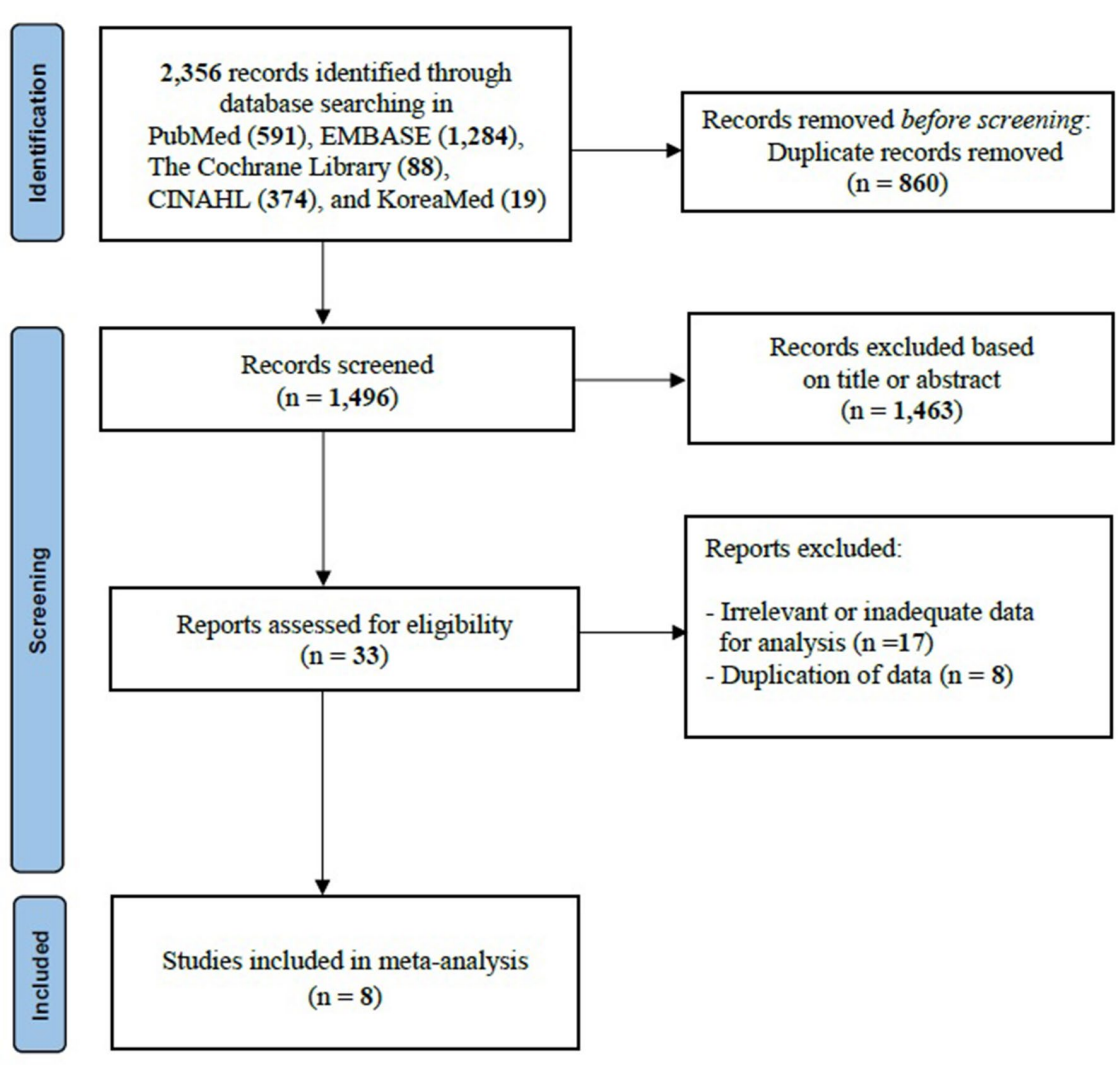
Flow diagram for study selection.

Of 2356 relevant records identified through the database search, 860 duplicated records were removed. After the screening process, two authors excluded 1463 articles from 1496 records based on the title and abstract (κ = 0.87). Thirty-three reports were left, and the eligibility assessment was done (κ = 1.00). Finally, eight (0.3%) met the inclusion criteria (κ = 1.00). The flow chart of the study selection process is shown in Fig. 1. Details of the study population, the definitions of PHT used in the analyzed studies, and the study outcomes are shown in Table 1. Among the eight studies, seven were cohort studies (three prospective and four retrospective studies), and one study was a case–control study. The results of the quality assessment are shown in Table 2. None of the studies had a high risk of bias; thus, all of them were included in the systematic review and meta-analysis.

**Table 1:** Characteristics of included studies.

First author, Year	Study design	Population	Exclusion criteria	Early PHT (%)	Definition of early PHT	Outcome measures	Definition of BPD
Berenz, 2017	Retrospective cohort study	Birthweight < 1500 g	Major CHD or congenital malformations with potential cardiopulmonary defects	23 (78/343)	echoCG at 72 h-14 PND any cardiac shunt with bidirectional or RL shunt increased TR velocity IVS flattening	BPD, in-hospital mortality	NICHD workshop, 2001
Kaluarachchi, 2018	Retrospective cohort study	GA 22^+0^–27^+6^ weeks	Death before 14 days, transferred into the NICU after 14 days of life or transferred out before 36 weeks PMA, multiple congenital anomalies, major CHD, no echoCG before 14 days of age	20 (31/154)	echoCG at 5–14 PND any cardiac shunt with bidirectional or RL shunt estimated RVSP > 40 mmHg RVSP/SSP > 0.5	BPD, in-hospital mortality, late PHT	Modified NIH workshop by removing the need for 28 days of supplemental oxygen
Kim, 2021	Retrospective cohort study	GA 22^+0^–27^+6^ weeks	Congenital malformations, no echoCG on postnatal 4–7 days	30 (74/247)	echoCG at 4–7 PND any cardiac shunt with bidirectional or RL shunt estimated RVSP > 40 mmHg IVS flattening	BPD, in-hospital mortality, late PHT	NICHD workshop, 2001
Mirza, 2014	Prospective cohort study	GA < 28 weeks	Major CHD, congenital pulmonary anomaly, congenital diaphragmatic hernia, death before the first study of echoCG	8 (10/120)	echoCG at 10–14 PND RVSP/SSP ≥ 0.5 IVS flattening	BPD, in-hospital mortality, late PHT	NICHD workshop, 2001
Mourani, 2015	Prospective cohort study	GA < 34 weeks and birthweight 500–1250 g	Major CHD, lethal congenital abnormality, anticipated death before hospital discharge	42 (115/277)	echoCG at 7 PND any cardiac shunt with bidirectional or RL shunt RVSP > 40 mmHg RVSP/SSP > 0.5 IVS flattening	BPD, late PHT	Modified NIH workshop with application of the oxygen reduction test
Seo, 2017	Retrospective cohort study	GA < 30 weeks	Major CHD, congenital pulmonary anomalies	16 (11/67)	echoCG < 14 PND or clinical findings RL shunt through the ductus IVS flattening	BPD, in-hospital mortality	NICHD workshop, 2001
Alvarez-Fuente, 2019	Prospective cohort study	GA < 28 weeks and birthweight ≤ 1250 g	Major congenital malformation, neurological lesion, mother with HIV	9 (19/47)	echoCG at 7 PND RSVP/SSP > 0.35	BPD	NICHD workshop, 2001
Seth, 2017	Retrospective case–control study	GA < 34 weeks	Chromosomal anomalies, major non-cardia congenital anomalies, CHD	3 (60/180)	echoCG or clinical findings < 14 PND any cardiac shunt with bidirectional or RL shunt estimated RVSP by TR velocity IVS flattening with moderate to severe right ventricular dysfunction	In-hospital mortality	Need for oxygen supplementation at 36 weeks (no evaluation on severity)

*PHT* pulmonary hypertension, *GA* gestational age, *CHD* congenital heart disease, *PMA* postmenstrual age, *HIV* human immunodeficiency virus, *echoCG* echocardiography, *PND* postnatal days, *RL* right-to-left, *TR* tricuspid regurgitation, *IVS* interventricular septum, *RVSP* right ventricular systolic pressure, *SSP* systemic systolic pressure, *BPD* bronchopulmonary dysplasia, *NICHD* National Institute of Child Health and Human Development, *NIH* National Institute of Health.

**Table 2:** Assessment of the risk of bias of included studies using a modified Newcastle–Ottawa scale.

Studies	Selection (max. 4)	Comparability (max. 2)	Outcome (max. 3)	Total score (max. 9)	Overall risk
Berenz, 2017	3	2	3	8	Low
Kaluarachchi, 2018	3	0	3	6	Moderate
Kim, 2021	3	1	3	7	Low
Mirza, 2014	4	0	3	7	Low
Mourani, 2015	4	2	3	9	Low
Seo, 2017	3	0	3	6	Moderate
Alvarez-Fuente, 2019	4	0	3	7	Low
Seth, 2017	2	2	2	6	Moderate

### Incidence of early pulmonary hypertension

**Figure 2:**
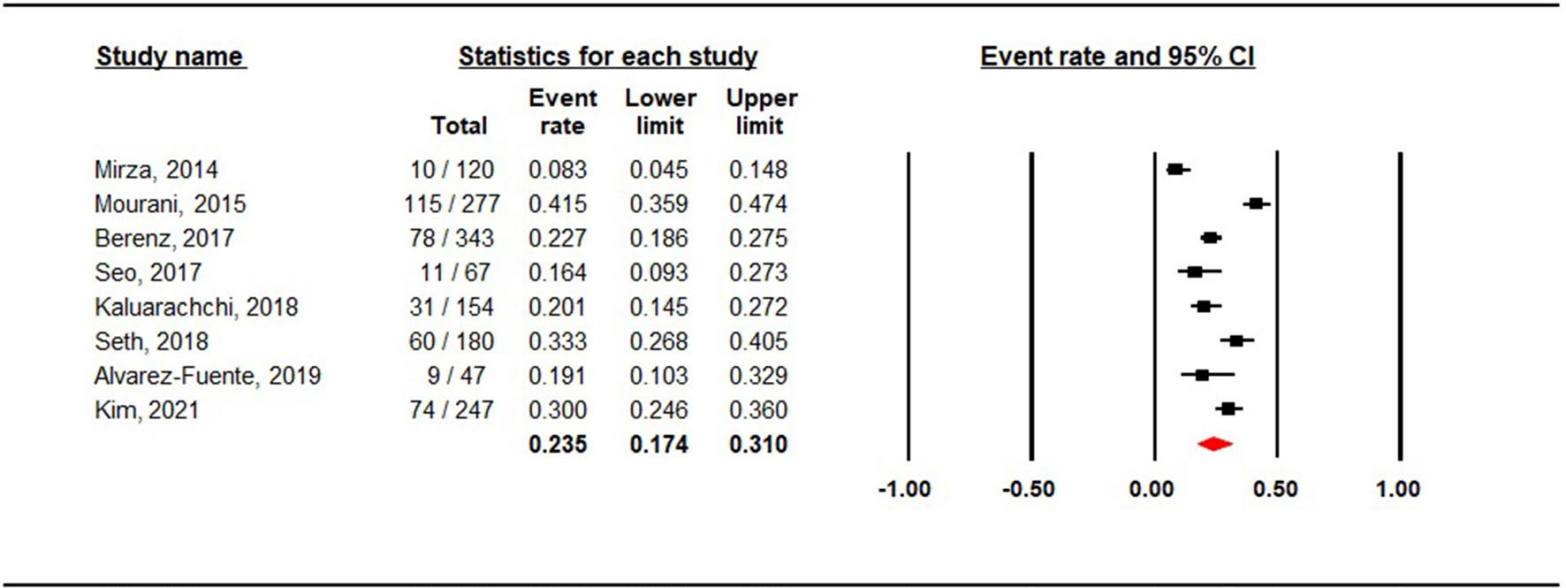
The rate of early pulmonary hypertension in preterm infants.

A total of 1435 preterm infants were included in the studies and 388 were reported to have early PHT. Analysis using a random-effects model showed that the overall event rate of early PHT was 24% (95% CI 0.174–0.310) (Fig. 2). Although heterogeneity was detected (*I*^2^ = 88.7%; *P* < 0.001), the sensitivity analysis showed stable results even after sequential exclusion of each study.

### Clinical outcomes of early pulmonary hypertension: BPD, mortality, and late PHT

**Figure 3:**
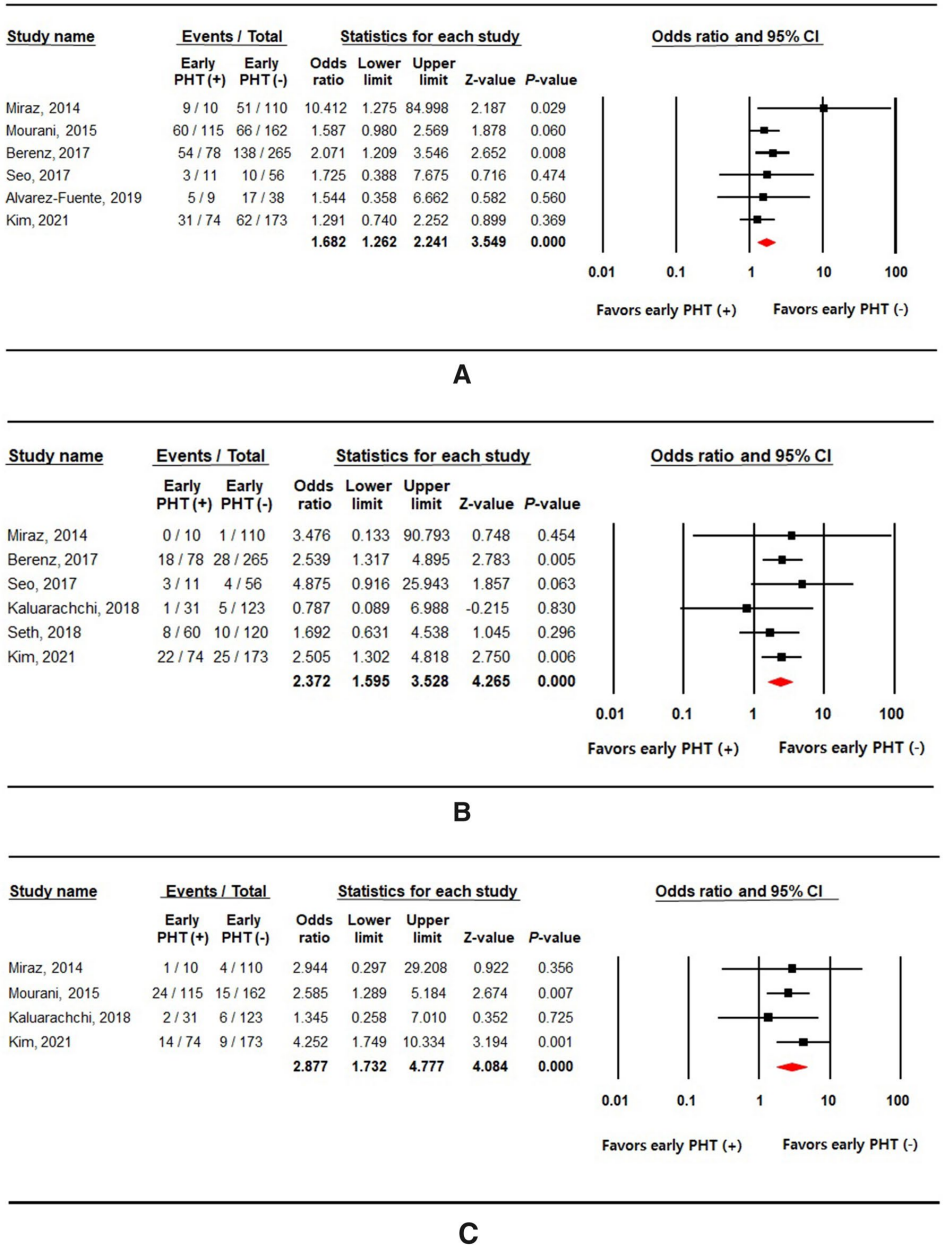
Forest plots of clinical outcomes associated with early pulmonary hypertension: (**A**) moderate to severe bronchopulmonary dysplasia at 36 weeks postmenstrual age, (**B**) in-hospital mortality, and (**C**) late pulmonary hypertension.

The incidence of moderate to severe BPD at 36 weeks postmenstrual age was reported in six studies (Fig. 3A), and it was significantly associated with early PHT. The overall OR was 1.682, indicating statistical significance (6 studies; 1101 subjects; 95% CI 1.262–2.241; *P* < 0.001; heterogeneity: *I*^2^ = 0%; *P* = 0.492). The sensitivity analysis showed stable results. The results of the funnel plot appeared to be asymmetrical (Supplementary Fig. S1A). However, the trim and fill method resulted in no additional imputed study, and suggests that small-study effects do not arise from publication bias.

In-hospital mortality was reported in six studies, whereas the number of deaths among infants with early PHT was not precisely described in two studies. Infants diagnosed with early PHT had an increased risk for in-hospital mortality (6 studies; 1111 subjects; OR 2.372; 95% CI 1.595–3.528; heterogeneity: *I*^2^ = 0%; *P* = 0.811; Fig. 3B). The results also showed that preterm infants with early PHT had an increased likelihood of developing late PHT (4 studies; 798 subjects; OR 2.877; 95% CI 1.732–4.777; heterogeneity: *I*^2^ = 0%; *P* = 0.648; Fig. 3C). The sensitivity analysis showed similar results for both outcomes. The funnel plots showed asymmetry (Supplementary Fig. S1B and S1C). We applied a trim and fill method to correct publication bias by adding one article in each analysis. However, the overall effects of early PHT on in-hospital mortality (pooled OR from the random-effects model, 2.272; 95% CI 1.544–3.343) and late PHT (pooled OR from the random-effects model, 3.113; 95% CI 1.917–5.056) were not either significantly reduced or increased after the adjustment for small-study effects.

### Risk factors related to early pulmonary hypertension

**Figure 4:**
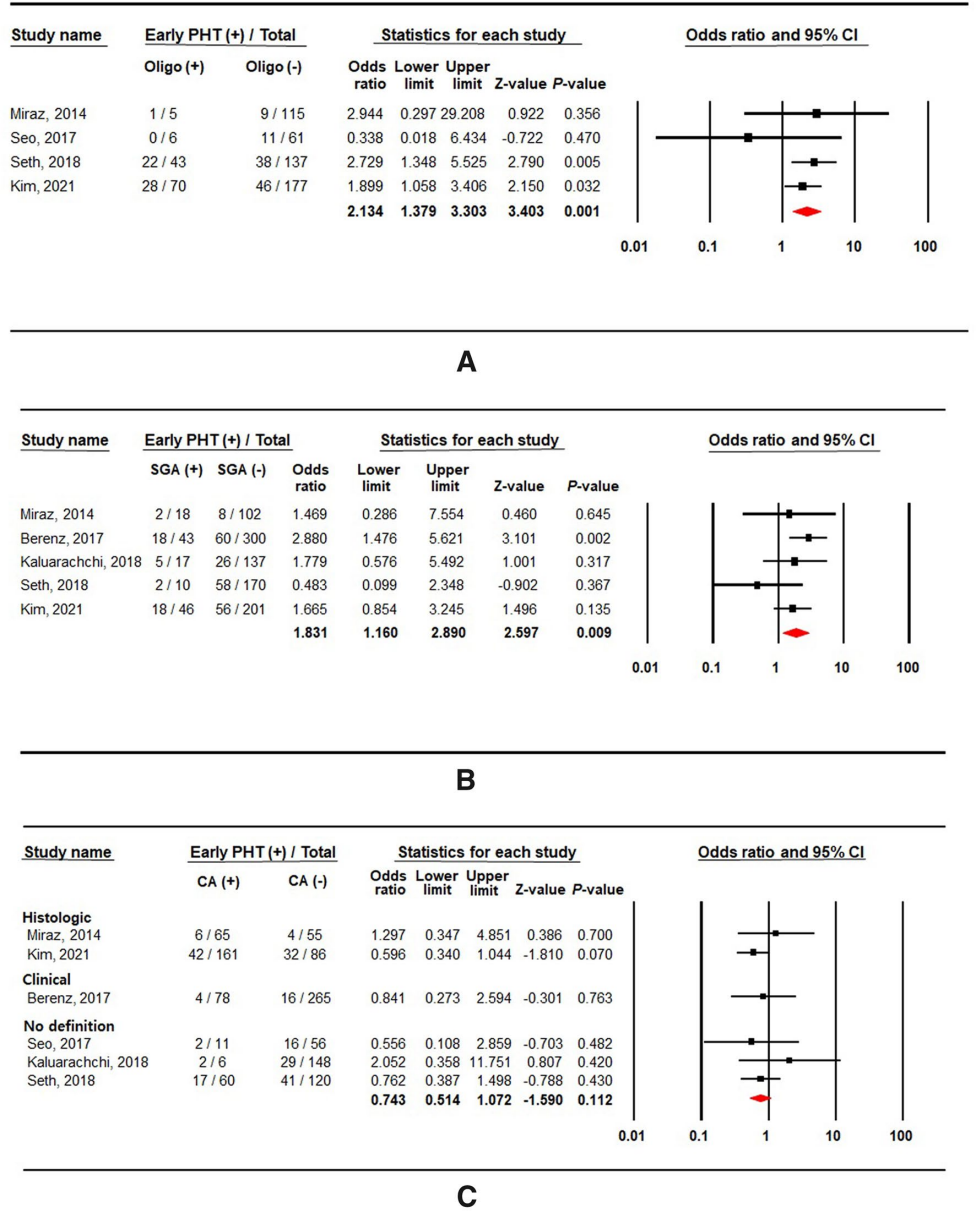

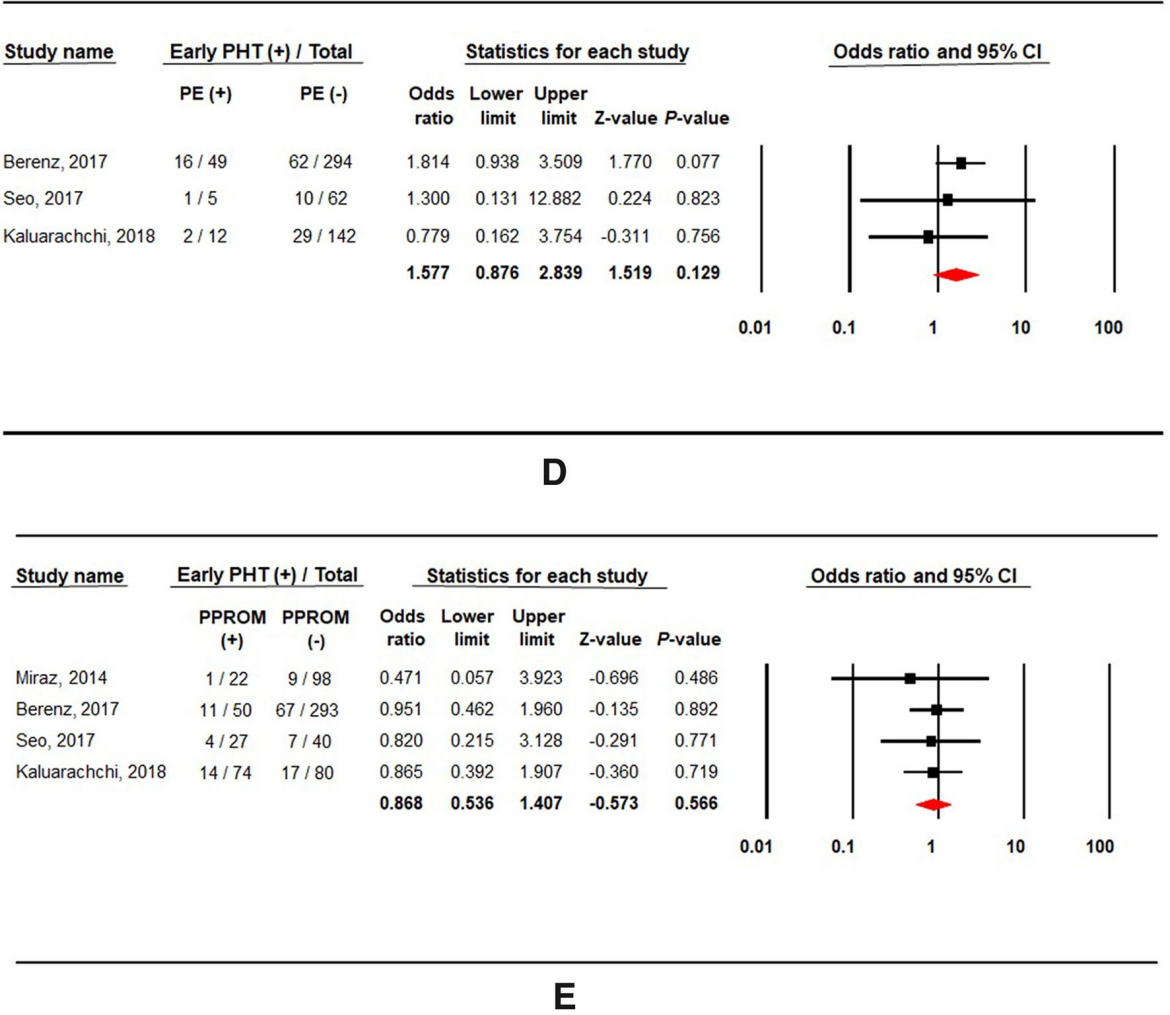
Forest plots of risk factors associated with early pulmonary hypertension: (**A**) oligohydramnios (Oligo), (**B**) small-for-gestational-age (SGA), (**C**) chorioamnionitis (CA), (**D**) preeclampsia (PE), and (**E**) preterm premature rupture of membrane (PPROM).

Oligohydramnios and SGA (birth weight < 10th percentile) were significantly associated with early PHT (Fig. 4A,B). The odds ratio was 2.134 for oligohydramnios (4 studies; 614 subjects; 95% CI 1.379–3.303; *P* = 0.001; heterogeneity: *I*^2^ = 0%; *P* = 0.532), and 1.831 for SGA (5 studies; 1044 subjects; 95% CI 1.160–2.890; *P* = 0.009; heterogeneity: *I*^2^ = 13%; *P* = 0.328). There was no significant change in the result after the sensitivity analysis was conducted by sequential exclusion of each study. Small-study effects were confirmed after visual inspection of the funnel plots for studies reporting oligohydramnios and SGA (Supplementary Fig. S2A,B). However, the result after the trim and fill method suggested that publication bias is less likely the cause of the funnel plot asymmetry in the meta-analysis of oligohydramnios. The contour-enhanced funnel plot of studies reporting SGA showed missing studies would be located on the right side in areas of statistical non-significance of the plot (Supplementary Fig. S2B). After applying the trim and fill method to adjust for small-study effects, the overall effect of SGA on early PHT was considerably increased (pooled OR from the random-effects model, 2.104; 95% CI 1.276–3.467).

Chorioamnionitis (6 studies; 1111 subjects; OR 0.743; 95% CI 0.514–1.072; *P* = 0.112; heterogeneity: *I*^2^ = 0%; *P* = 0.738; Fig. 4C) and preeclampsia (3 studies; 564 subjects; OR 1.577; 95% CI 0.876–2.839; *P* = 0.129; heterogeneity: *I*^2^ = 0%; *P* = 0.615; Fig. 4D) were not associated with the risk of early PHT. Both histological and clinical chorioamnionitis were reported in one study^13^, whereas each of them was described in two other studies^15,20^. Neither clinical nor histological chorioamnionitis showed any significant association with early PHT when stratified according to the definition of chorioamnionitis. Four studies provided PPROM history, and 30 preterm infants had PPROM among 130 (23%) infants with early PHT. No significant association was observed between PPROM and early PHT (OR 0.868; 95% CI 0.536–1.407; *P* = 0.566; heterogeneity: *I*^2^ = 0%; *P* = 0.943; Fig. 4E).

## Discussion

In this systematic review and meta-analysis, we investigated the incidence and risk factors of early PHT in preterm infants. In addition, various aspects of early PHT, including perinatal factors and in-hospital outcomes, were analyzed. The results showed that early PHT occurred in 24% of preterm infants. Oligohydramnios and SGA were the perinatal factors associated with early PHT. In addition, moderate to severe BPD, in-hospital mortality, and late PHT were associated with early PHT in the study population.

PHT is considered one of the phenotypes of pulmonary vascular disease in premature neonates. However, certain clinical courses, such as perinatal asphyxia and respiratory distress syndrome, could interfere with the postnatal transition of newborn infants, leading to delay in decreased pulmonary vascular resistance rather than pulmonary vascular disease itself^21,22^. For instance, PHT diagnosed within 72–96 h of life could be defined as delayed postnatal cardiopulmonary adaptation, whereas subsequent PHT within 5–14 days of life could be defined as early PHT in preterm infants^23^. In the present review, we analyzed studies that included preterm infants with clinical symptoms of PHT, as well as relevant echocardiographic findings, within 2 weeks of age. In six studies, early PHT was diagnosed using echocardiography performed at least 72 h after birth^12,13,15,20,24,25^ to minimize the inclusion of cases of delayed pulmonary vascular transition. However, in the studies by Seth et al. and Seo et al., early PHT was diagnosed within 3 days after birth based on clinical symptoms and echocardiographic findings^14,26^. Even after excluding these two studies that included neonates with early PHT diagnosed within 3 days after birth, the results of the present study remained consistent with those of previous studies.

SGA and oligohydramnios, which are strong predictors of BPD in preterm infants^27–30^, were associated with early PHT in the present study. Although the association between BPD and PHT has been recently highlighted, the fetal mechanisms that contribute to BPD and PHT are poorly understood. Several studies have indicated that intrauterine growth restriction is associated with impairment of the vascular endothelial growth factor and nitric oxide signaling pathways, which lead to PHT after birth^22,31,32^. Oligohydramnios, followed by premature rupture of the membrane, could also disrupt the development of a premature lung, leading to PHT in the early neonatal period^33^. Reduction in the size of the pulmonary vascular bed, decreased vessel count, and enhanced proliferation of the pulmonary arterial smooth muscle cells are observed following a period of prolonged oligohydramnios^3,34^. Regarding late PHT, a previous systematic review and meta-analysis showed that oligohydramnios and SGA are associated with BPD-associated PHT^35^. Interestingly, SGA and oligohydramnios have been reported as factors associated with a spectrum of pulmonary vascular diseases, such as early PHT, BPD, and late PHT.

Regarding neonatal outcomes, BPD, late PHT, and mortality were also associated with early PHT in the present study. Structural damages could result from pulmonary vascular disease and early PHT, leading to BPD. Moreover, persistent exposure to hypoxia, hyperoxia, or hemodynamic stress in patients with BPD can induce remodeling of the pulmonary arteries and lead to late PHT with increased pulmonary vascular resistance. These clinical conditions may be understood as a spectrum of pulmonary vascular diseases. However, as they occur late in the neonatal period, these conditions could be influenced by hypoxia, hemodynamic stress, infection, or inflammation, which commonly occur in preterm infants during the postnatal period^36^. Therefore, the association between early PHT and BPD observed in the present study should be interpreted with caution since multiple factors are involved in the pathogenesis of BPD. Several studies have reported preterm infants with early PHT needed more ventilator support^13–15,20^, and lung injury due to prolonged ventilator care may have caused BPD. On the contrary, strategies to reduce BPD, such as fluid restriction management in the first week of life to minimize secondary lung injury due to significant patent ductus arteriosus or non-invasive respiratory support, may decrease the outcome. Maternal and postnatal confounding factors, such as chorioamnionitis, preeclampsia, patent ductus arteriosus, and sepsis, could also contribute to the development of BPD. However, owing to the limitation of available data, we could not perform a meta-analysis considering these factors. Additional research controlling for these confounding factors is needed to better understand the relationship between early PHT and BPD.

Cardiac catheterization is regarded as the gold standard for diagnosing PHT. Because of its invasive nature, an echocardiogram is used as an alternative for diagnosing PHT in neonates. Estimated RVSP or RVSP/systolic systemic pressure ratio, interventricular septal flattening, and direction of shunt flow are usually considered qualitative and quantitative echocardiographic variables of PHT. However, classification of the severity using these parameters is not consistently reported in studies. In addition, specific guidelines for the screening and follow-up of early PHT using echocardiography have not been established yet. The pediatric PHT guidelines released by the American Heart Association and American Thoracic Society recommend using echocardiography to evaluate PHT in all infants with established moderate or severe BPD at 36 weeks of age^37^. However, the guidelines do not include recommendations for early PHT. In addition, most of the studies included in the analysis were retrospective studies. Therefore, prospective multicenter studies are needed to establish a protocol for the interpretation of echocardiograms of early PHT.

As far as we know, this is the first study that provides clinical outcomes and associated risk factors of early PHT diagnosed within 2 weeks of age in preterm infants. However, there are several limitations in the present study. First, the association between SGA and early PHT should be interpreted with caution. Although the overall effect of SGA on early PHT was statistically significant, the funnel plot suggested the possibility of publication bias. After adjusting small-study effects using the trim and fill method, the risk of SGA was considerably higher, which implies publication bias. Still, born SGA has the same effect on the development of early PHT regardless of publication bias. Second, due to limitations of available data, there have been several changes in our analysis compared to the PROSPERO protocol. Subgroup analysis comparing outcomes between preterm infants born below and after 28 weeks’ gestation was not feasible because all of the studies included extremely preterm births. In addition, articles had different definitions of morbidities, such as late-onset sepsis or necrotizing enterocolitis; therefore, analyzing these outcomes was unattainable.

Despite these limitations, the findings of the present study suggest the importance of evaluating early PHT in preterm infants within 2 weeks of age. Preterm infants who are SGA or exposed to oligohydramnios will benefit from early screening and continuous monitoring of PHT using echocardiography.

## Supplementary Information

Supplementary Information 1.

Supplementary Figure 1.

Supplementary Figure 2.

## Data Availability

The data that support the findings of the current study are available from the corresponding author on reasonable request.
