# Supplementary material for: Risk factors of early pulmonary hypertension and its clinical outcomes in preterm infants: a systematic review and meta-analysis
Source: Sci Rep. 2022 Aug 19;12:14186. doi: 10.1038/s41598-022-18345-y (PMC9391329; doi:10.1038/s41598-022-18345-y)
Supplement: Supplementary file 3 — Supplementary Figure 2. [file 41598_2022_18345_MOESM3_ESM.docx]

**Supplementary Figure S2. Contour-enhanced funnel plots for detecting publication bias of pooled association between early pulmonary hypertension and (A) oligohydramnios and (B) small-for-gestational- age.** Filled circle indicates included study, while open circle suggests imputed study identified from the trim and fill method.

**(A)
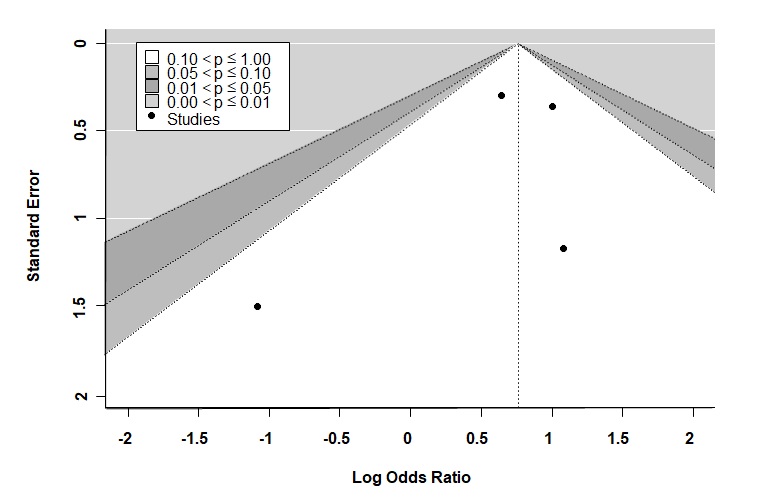
**

**(B)**

**
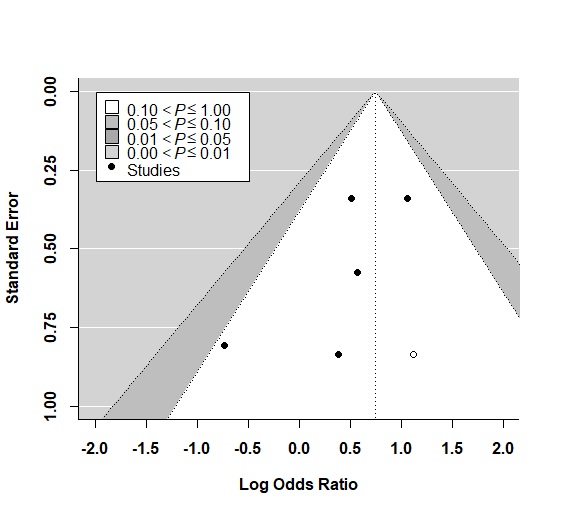
**
